# CAML: Commutative Algebra Machine Learning—A Case Study on Protein–Ligand Binding Affinity Prediction

**DOI:** 10.1021/acs.jcim.5c00940

**Published:** 2025-06-16

**Authors:** Hongsong Feng, Faisal Suwayyid, Mushal Zia, JunJie Wee, Yuta Hozumi, Chun-Long Chen, Guo-Wei Wei

**Affiliations:** † Department of Mathematics and Statistics, University of North Carolina at Charlotte, Charlotte, North Carolina 28223, United States; ‡ Department of Mathematics, King Fahd University of Petroleum and Minerals, Dhahran 31261, KSA; § Department of Mathematics, 3078 Michigan State University, East Lansing, Michigan 48824, United States; ∥ Department of Physiology, Michigan State University, East Lansing, Michigan 48824, United States; ⊥ Physical Sciences Division, 6865 Pacific Northwest National Laboratory, Richland, Washington 99354, United States; # Department of Biochemistry and Molecular Biology Michigan State University, East Lansing, Michigan 48824, United States; ∇ Department of Electrical and Computer Engineering Michigan State University, East Lansing, Michigan 48824, United States

## Abstract

Recently, Suwayyid and Wei introduced commutative algebra as an emerging paradigm for machine learning and data science. In this work, we propose commutative algebra machine learning (CAML) for the prediction of protein–ligand binding affinities. Specifically, we apply persistent Stanley–Reisner theory, a key concept in combinatorial commutative algebra, to the affinity predictions of protein–ligand binding and metalloprotein–ligand binding. We present three new algorithms, i.e., element-specific commutative algebra, category-specific commutative algebra, and commutative algebra on bipartite complexes, to tackle the complexity of data involved in (metallo) protein–ligand complexes. We show that the proposed CAML outperforms other state-of-the-art methods in (metallo) protein–ligand binding affinity predictions, indicating the great potential of commutative algebra learning.

## Graphical abstract

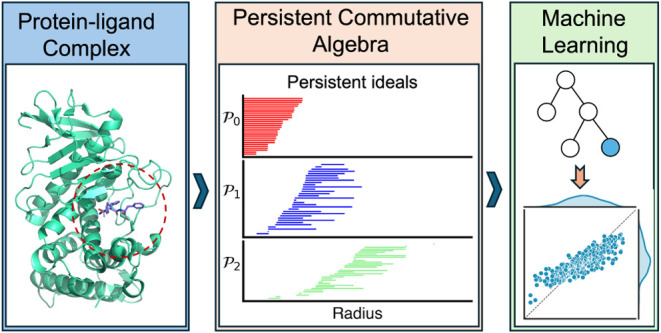

## Introduction

Drug discovery plays a vital role in contemporary medicine, profoundly impacting global health outcomes. Conventional drug development processes, however, are time-intensive and costly, requiring more than a decade and billions of dollars to commercialize a single drug.^1^ Established techniques such as molecular docking,^2–5^ free energy perturbation,^6^ and empirical modeling^7^ have propelled advancements but face inherent constraints. These methods often suffer from inaccuracies, demand substantial computational resources for large-scale analyses, and may overlook novel binding sites or interaction dynamics, potentially missing therapeutic breakthroughs.

Machine-learning (ML) approaches are gaining traction as powerful tools in drug design,^8–11^ renowned for their capacity to forecast protein structures and detect intricate patterns for enhanced predictions.^12^ The adoption of deep learning, integrated with chemoinformatics and bioinformatics,^13^ marks a transformative shift toward data-driven methodologies in pharmaceutical research.^14–16^ Nevertheless, challenges persist, including limited data sets,^17^ data imbalance,^18^ intricate molecular architectures, and stereochemical complexities. Furthermore, embedding essential physical interactions, such as hydrogen bonding, van der Waals forces, hydrophobic effects, electrostatic forces, and ionic bonds, into ML algorithms for protein–ligand binding remains a significant hurdle.^19–21^

To address these limitations, researchers are employing sophisticated mathematical frameworks rooted in algebraic topology, differential geometry, and combinatorial graph theory.^22^ These multiscale models, previously successful in characterizing biomolecular systems,^23–27^ capture fundamental physical, chemical, and biological interactions critical to protein–ligand binding while clarifying the three-dimensional (3D) structural intricacies of these complexes. Notably, these approaches have delivered top-tier performances in the D3R Grand Challenges, a leading international competition in computer-aided drug design.^24,28^ Inspired by this success, there is a continuous effort in computational biology and applied mathematics to seek advanced mathematical representations of complex biomolecules such as proteins and their interactions.

Commutative algebra is a branch of mathematics that studies commutative rings, their ideals, modules, and related structures.^29,30^ It serves as a foundational framework for algebraic geometry, number theory, and many other areas of mathematics. Its key concepts include Noetherian rings, Cohen-Macaulay rings, localization theory, primary decomposition, dimension theory, and homological algebra.

Despite its importance in pure mathematics, it has hardly been applied in data science and artificial intelligence. Recently, Suwayyid and Wei introduced persistent Stanley–Reisner theory to bridge commutative algebra, algebraic topology, machine learning, and data science.^31^ Stanley–Reisner theory is the study of the commutative algebra, i.e., square-free monomial ideals in a polynomial ring, of simplicial complexes, structured sets comprising points, line segments, triangles, and their higher-dimensional counterparts.^32–34^ Therefore, persistent Stanley–Reisner theory (PSRT) enables commutative algebra analysis (CAA) of point cloud data and machine-learning predictions. Specifically, PSRT examines how the Stanley–Reisner structure of a complex evolves under filtration. Many computable quantities, including persistent graded Betti numbers via Hochster’s formula, persistent *f*-vectors, persistent *h*-vectors, and persistent facet ideals, have been proposed. Facet persistence barcodes, which record the birth and death of persistent facet ideals as the simplicial complex evolves, have been introduced for practical applications in data science. PSRT provides novel insights into geometry, topology, and combinatorial techniques at multiple scales. An important motivation for this development was persistent homology,^35,36^ an algebraic topology tool for topological data analysis (TDA) and topological deep learning (TDL),^37^ a new frontier for relational learning.^38^

The objective of this work is to explore the utility and demonstrate the potential of commutative algebra machine learning (CAML) for protein–ligand binding affinity prediction. We consider two benchmark data sets: the PDBbind-v2016 data set for protein–ligand binding interactions^39^ and a metalloprotein–ligand binding data set.^40^ As these data sets involve intricate three-dimensional (3D) protein–ligand complexes as well as complex physical and chemical interactions, we propose a few new CAML algorithms, i.e., element-specific commutative algebra, category-specific commutative algebra, and commutative algebra on bipartite complexes, to capture intrinsic physical and chemical interactions, such as hydrogen bonding, van der Waals forces, hydrophobic effects, electrostatic forces, and ionic bonds in 3D metalloprotein–ligand complexes. As shown in the results, the proposed CAML consistently outshines its peers, achieving state-of-the-art outcomes across benchmark data sets in protein–ligand binding affinity prediction.

The rest of this article is organized as follows. Results section is devoted to the results of CAML for protein–ligand binding and metalloprotein–ligand binding predictions. Methods are described in Methods section. This work ends with a conclusion.

## Results

### Protein–Ligand Binding Affinity Predictions

The PDBbind database^39^ is a widely recognized, curated resource that systematically collects experimentally determined 3D structures of protein–ligand complexes alongside their corresponding binding affinity data, e.g., dissociation constants *K*_d_, inhibition constants *K*_*i*_, and Gibbs free energy changes Δ*G*. It serves as a gold-standard benchmark for developing and validating computational models aimed at predicting protein–ligand binding affinities (BAs).

Leveraging our persistent Stanley-Rensiner theory (PSRT), we developed commutative algebra machine-learning (CAML) models to predict protein–ligand binding affinities. The models were benchmarked against established methods using the widely recognized PDBbind data set. Specifically, we focused on the PDBbind-v2016 data set, a rigorously curated version with clearly defined training (3768 complexes) and test sets (290 complexes). The PDBbind database^39^ provides a comprehensive collection of 3D protein–ligand structures paired with binding affinity data. As shown in Figure 1a, our PSRT-guided model, CAML, outperformed existing state-of-the-art approaches, achieving superior predictive accuracy in the binding affinity estimation.

**Figure 1:**
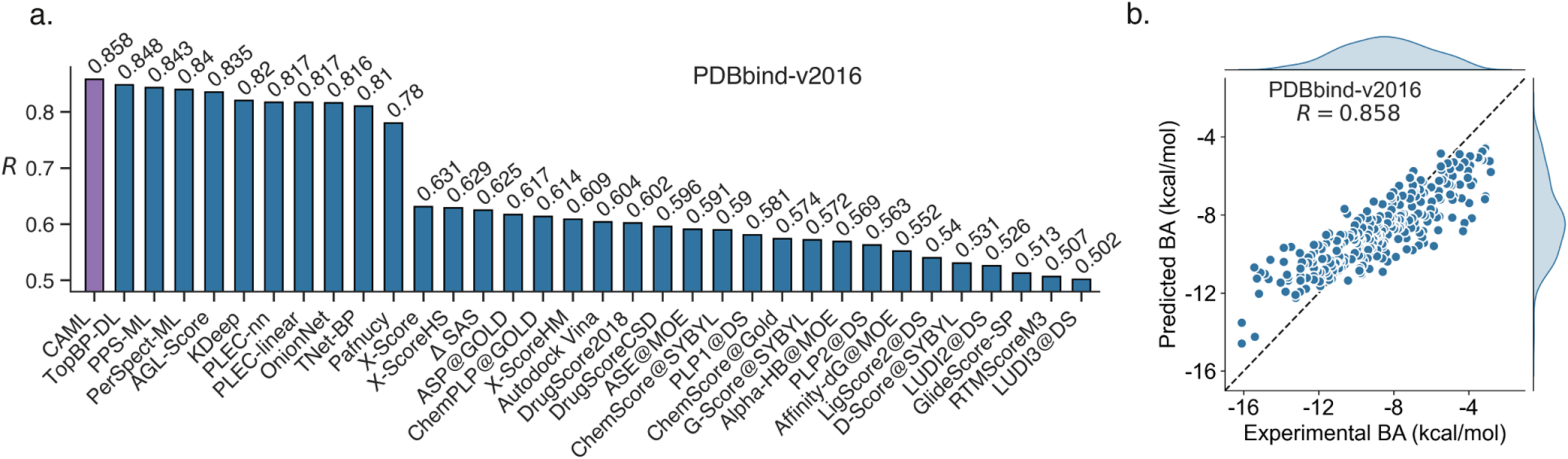
(a) Comparison of the predictions from our CAML model with other published models in terms of Pearson correlation coefficient (PCC) (*R*) on the PDBbind-v2016 data set. (b) Comparison between the experimental and predicted binding affinities (BAs) from our CAML model for the PDBbind-v2016 data set.

Numerous competitive models rooted in mathematical or physical frameworks,^19–21^ such as persistent homology^23^ and persistent spectral theories,^26,41^ have been reported. These rank among the top performers in this domain (Figure 1a). On the PDBbind-v2016 test set, CAML achieved a Pearson correlation coefficient (*R*) of 0.858, significantly surpassing persistent homology-based TopBP-DL (*R* = 0.848)^23^ and persistent spectral theory-based models PerSpect-ML (*R* = 0.843)^26^ and PPS-ML (*R* = 0.840).^41^ Several competitive neural network-based models for protein–ligand binding affinity prediction have also been benchmarked on the PDBbind-v2016 test set.^42–44^ We conducted extensive predictive comparisons on this data set with models from the literature, as shown in Figure 1a, which highlights the superior performance of our model in protein–ligand binding affinity prediction. These results highlight CAML’s efficacy as a novel analytical tool and its ability to drive advanced predictive models for binding affinity.

CAML’s reliability is further demonstrated by the strong alignment between experimental and predicted binding affinities, as visualized in Figure 1b. Its success stems from three key innovations: (1) PSRT-driven molecular data analysis, (2) element-specific (ES) and category-specific (CS) modeling of intra- and intermolecular interactions, and (3) integration of natural language processing (NLP) via a transformer architecture (details in Vectorization of Persistent Commutative Algebra section).

As detailed in Table 1, we evaluated five distinct models. The top performer (final CAML model, *R* = 0.858) combines consensus predictions from CAML­(ES, CS) (a fusion of element- and category-specific strategies) with transformer-based sequence analysis. The ES approach, a widely adopted method for dissecting atomic interactions, and the CS strategy, which categorizes interactions by atomic properties, were individually effective. Their combination (CAML­(ES, CS), *R* = 0.853) further enhanced the accuracy. Integrating these with sequence-based NLP predictions via the transformer model yielded the final CAML’s performance, highlighting the synergy between structural and sequential pattern analysis.

**Table 1:** Modeling Performance of Various Strategies on the Test Set of PDBbind-v2016^a^

data set	CAML(ES)	CAML(CS)	CAML(ES,CS)	transformer	CAML(ES,CS) + transformer
*R*	0.836 ± 0.001	0.834 ± 0.001	0.845 ± 0.001	0.836 ± 0.001	**0.858 ± 0.001**
RMSE	1.743 ± 0.004	1.745 ± 0.004	1.719 ± 0.003	1.713 ± 0.005	**1.669 ± 0.004**

a The evaluation metrics used are the pearson correlation coefficient (*R*) and the root mean square error (RMSE, in kcal/mol). Twenty independent runs with different random seeds were performed, and the average metric values are reported. The ± sign indicates the standard deviation value. CAML­(ES) and CAML­(CS) refer to commutative algebraic machine-learning models combined with element-specific and category-specific atom combinations, respectively. CAML­(ES, CS) represents the consensus results from the CAML­(ES) and CAML­(CS) Models. Transformer refers to sequence-based modeling using natural language processing. CAML­(ES, CS) + transformer indicates the consensus predictions from the CAML­(ES, CS) and transformer models, which is defined as our final CAML model.

This multifaceted approach positions CAML as a state-of-the-art tool for protein–ligand binding affinity prediction, with implications for drug discovery and molecular design.

### Metalloprotein–Ligand Binding Affinity Predictions

As another benchmark example, we consider metalloprotein–ligand binding affinities. Metalloproteins are proteins that incorporate metal ions as integral structural components and play indispensable roles in biological processes such as cellular respiration, electron transfer, catalytic reactions, and structural stabilization.^45–47^ More specifically, protein-metal-binding sites are responsible for catalyzing some of the most difficult yet important functions, such as photosynthesis, respiration, water oxidation, molecular oxygen reduction, and nitrogen fixation. Studies estimate that roughly half of all proteins in biology are metalloproteins.^48–50^ The prediction of metalloprotein–ligand binding affinities represents a critical challenge in drug discovery. Deciphering the structure, such as function relationships and interaction mechanisms, of metalloproteins is pivotal for unraveling fundamental biological pathways and accelerating the design of targeted therapeutics.

Recent advancements have addressed the scarcity of specialized data sets for this task. For example, the study by ref 40 introduced the largest curated data set to date for metalloprotein–ligand binding affinity prediction, providing a robust foundation for developing and benchmarking computational models in this domain.

Using the data set from,^40^ we constructed two CAML machine-learning models based on element-specific (ES) and category-specific (CS) strategies, resulting in CAML­(ES) and CAML­(CS) models. Figure 2a gives the comparisons between our CAML models with other published models in terms of the Pearson correlation coefficient (*R*). The previous state-of-art model is the JPH-GBT model,^51^ which gave a much higher *R* value than other models.^40,52,53^ Our CAML models redefines the state-of-art for metalloprotein–ligand binding affinity predictions. Model CAML­(ES) and CAML­(CS) give *R* values of 0.745 and 0.755, respectively. Figure 2b shows the comparison between the experimental binding affinity values and the predicted binding affinity using our CAML­(CS) model. Table 2 gives comparisons of our models with others in terms of *R* and RMSE metrics.

**Table 2:** Comparison of Our CAML Models with Existing Machine-Learning Approaches in Modeling Metalloprotein-Ligand Binding Affinity Data Set^a^

machine-learning models	*R*	RMSE
RosENet^40,52^	0.615 ± 0.017	1.436 ± 0.011
NNScore2.0 ^40,53^	0.629 ± 0.002	1.391 ± 0.004
MetalProGNet^40^	0.703 ± 0.010	1.285 ± 0.020
JPH-GBT^51^	0.742 ± 0.001	1.205 ± 0.001
CAML(ES)	0.745 ± 0.001	1.202 ± 0.002
CAML(CS)	**0.755** ± **0.001**	**1.185** ± **0.002**

a Two evaluation metrics, including pearson correlation coefficient (*R*) and root-mean-squared error (RMSE) are used. The ± sign indicates the standard deviation value. CAML­(ES) and CAML­(CS) refer to models utilizing the PSRT vectorization framework combined with element-specific (ES) and category-specific (CS) atom groupings, respectively. The RMSE values are computed based on the raw p*K*_d_ labels that serve as binding affinities.

**Figure 2:**
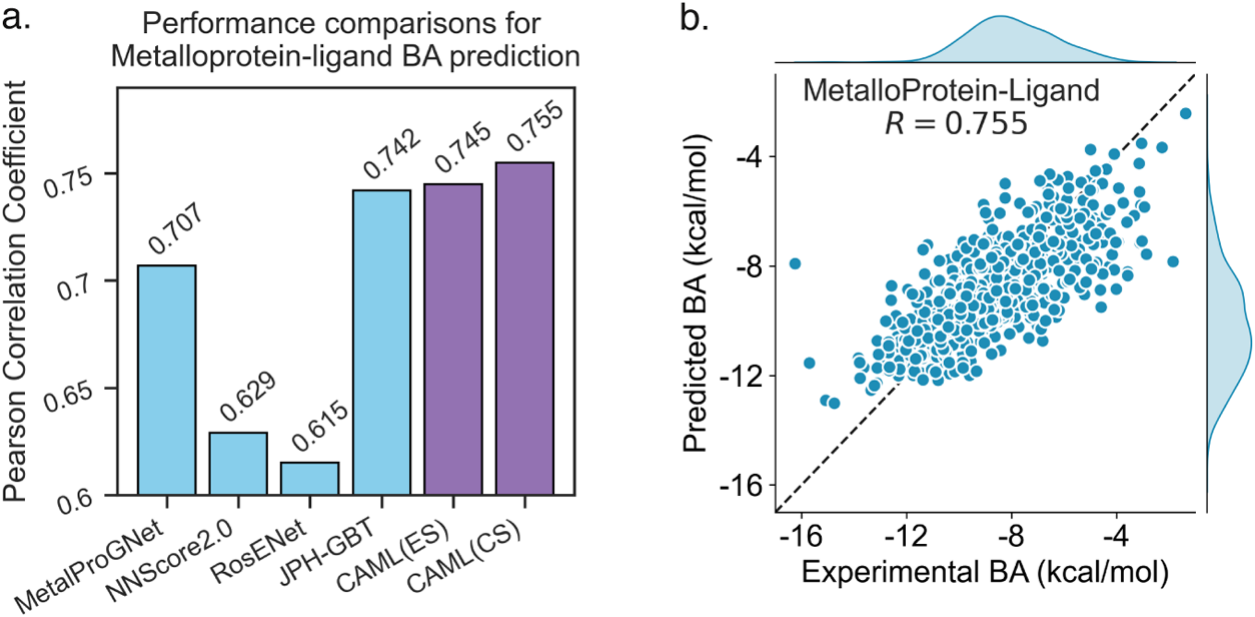
(a) Prediction performance of our CAML models on the metalloprotein–ligand binding affinity (BA) data set in terms of Pearson correlation coefficient (*R*). (b) Comparison between the experimental binding affinities and the predicted values from our CAML­(CS) model for this data set.

## Methods

In this section, we first list our data sets, showing the clear separation between training and test sets. Then, we provide an overview of the persistent Stanley–Reisner theory (PSRT). Next, we describe the vectorization of persistent commutative algebra followed by natural language processing (NLP) molecular descriptors. Machine-learning models and model parameters are given. We also define the evaluation metrics.

### Data Sets

The first benchmark data set we use is PDBbind-v2016, which is the largest protein–ligand binding affinity prediction data set with well-defined training and test sets. The second one is the metalloprotein–ligand data set compiled from PDBbind-v2020 in work.^40^ This original data set consists of training, validation, and test sets with different types of metal ions. We utilize their training and test sets to benchmark the performance of PSRT-based machine-learning models. Details of the dataset sizes are listed in Table 3.

### Persistent Stanley–Reisner Theory

Persistent Stanley–Reisner theory is a novel framework for analyzing the shape of data by leveraging tools from combinatorial commutative algebra.^31^ It encodes point cloud data as simplicial complexes—combinatorial structures built from vertices, edges, triangles, and higher-dimensional simplices—capturing both topological and combinatorial features inherent in the data. A filtration process is then applied to these complexes to track the evolution and persistence of such features across multiple spatial or geometric scales. This approach introduces algebraic invariants such as persistent *h*-vectors, *f*-vectors, graded Betti numbers, and facet ideals, thus providing a new algebraic perspective within the broader framework of topological data analysis.

#### Simplicial Complex

A simplicial complex Δ on the finite vertex set *V* = {*x*_1_, *x*_2_, ···, *x*_*n*_} is a collection of subsets of *V*, referred to as faces or simplices, satisfying the following conditions:

1. If *F* ∈ Δ and *G* ⊆ *F*, then *G* ∈ Δ.
2. For each *i* = 1, ···, *n*, the singleton {*x*_*i*_} belongs to Δ. In particular, every vertex is included as a face.

A face consisting of *r* + 1 vertices is called an *r*-dimensional face. The dimension of Δ is defined as the maximum dimension among its faces. A face that is maximal with respect to inclusion is called a facet of Δ, and the set of all such facets is denoted by F(Δ) .

#### Stanley–Reisner Theory and Facet Ideals

Let *k* be a field, and consider the standard polynomial ring

1 $$S=k\left[x_{1},x_{2},...,x_{n}\right]$$

endowed with the natural Z -grading determined by deg­(*x*_*i*_) = 1 for all *i* = 1, ···, *n*. Let Δ be a simplicial complex on the vertex set *V* = {*x*_1_, *x*_2_, ···, and *x*_*n*_}. The Stanley–Reisner ideal associated with Δ is defined as

2 $$I\left(\Delta \right)=\left\langle x_{i_{1}}x_{i_{2}}\cdot \cdot \cdot x_{i_{r}}|\left\{x_{i_{1}},x_{i_{2}},...,x_{i_{r}}\right\}\notin \Delta \right\rangle$$

that is, it is the ideal generated by all squarefree monomials corresponding to nonfaces of Δ. The quotient ring

3 k[Δ]=S/I(Δ)

is called the Stanley–Reisner ring of Δ.

It is a classical result that the Krull dimension of the Stanley–Reisner ring is given by

4 dim(k[Δ])=dim(Δ)+1

which we denote by *d*. Thus, the simplicial complex Δ is said to be (*d* – 1)-dimensional.

Now, for any subset *A* ⊆ *V* = {*x*_1_, *x*_2_, ···, *x*_*n*_}, we define the associated prime monomial ideal by

$$P_{A}≔\left(x_{i}|x_{i}\notin A\right)$$

In the context of Stanley–Reisner theory, we are particularly interested in the prime monomial ideals associated with the facets of Δ, referred to as the facet prime monomial ideals, or simply, facet ideals.

A fundamental property of the Stanley–Reisner ideal is that it admits a primary decomposition as the intersection of the facet ideals:

$$I\left(\Delta \right)=\underset{\sigma \in F\left(\Delta \right)}{\cap }P_{\sigma }$$

where F(Δ) denotes the set of all facets of the simplicial complex Δ.

#### Graded Betti Numbers, *f*-Vectors, and *h*-Vectors

As a graded *S*-module, *k*[Δ] admits a minimal free resolution of the form

5 $$\cdot \cdot \cdot \to \underset{j}{⊕}S{\left(-j\right)}^{\beta_{i,j}\left(k\left[\Delta \right]\right)}\to \cdot \cdot \cdot \to \underset{j}{⊕}S{\left(-j\right)}^{\beta_{0,j}\left(k\left[\Delta \right]\right)}\to k\left[\Delta \right]\to 0$$

where *S*(−*j*) is the graded free module *S* shifted in degree by *j* and graded Betti numbers are

6 $$\beta_{i,j}\left(k\left[\Delta \right]\right)=\dim_{k}{\mathrm{Tor}}_{i}^{S}{\left(k\left[\Delta \right],k\right)}_{j}$$

with Tor_*i*_^*S*^(*k*[Δ], *k*)_*j*_ being the Tor module, which measures how nontrivial the resolution is at homological degree *i*. For a subset *W* ⊆ *V*, the restriction (or induced subcomplex) of Δ to *W* is

7 $$\Delta_{W}=\left\{\tau \in \Delta :\tau \subseteq W\right\}$$

Hochster’s formula provides an explicit description of the Z -graded Betti numbers β_*i*,*j*+i_(*k*[Δ]) of the Stanley–Reisner ring *k*[Δ] in terms of the reduced simplicial homology of induced subcomplexes. For integers *i*, *j* ≥ 0, it states that

8 $$\beta_{i,j+i}\left(k\left[\Delta \right]\right)=\sum_{\begin{array}{l} W\subseteq \left\{x_{1}\;,...,x_{n}\;\right\}\\ \left|W\right|=j+i \end{array}}\dim_{k}{\mathrm{H̃}}_{j-1}\left(\Delta_{W};k\right)$$

where Δ_*W*_ denotes the subcomplex of Δ induced on the vertex set *W*, and *H̃*_*j*–1_(Δ_*W*_; *k*) is the (*j* – 1)-st reduced homology group with coefficients in *k*. This formula holds for 1 ≤ *i* ≤ *n* – 1 and 1 ≤ *j* ≤ min {*n* – *i*, dim­(Δ) + 1}.

In particular, for *j* = 1, the formula simplifies to

9 $$\beta_{i,i+1}\left(k\left[\Delta \right]\right)=\sum_{\begin{array}{l} W\subseteq \left\{x_{1}\;,...,x_{n}\;\right\}\\ \left|W\right|=i+1 \end{array}}\left(\beta_{0}\left(\Delta_{W}\right)-1\right)$$

and for *j* ≥ 2, it takes the form

10 $$\beta_{i,j+i}\left(k\left[\Delta \right]\right)=\sum_{\begin{array}{l} W\subseteq \left\{x_{1}\;,...,x_{n}\;\right\}\\ \left|W\right|=j+i \end{array}}\beta_{j-1}\left(\Delta_{W}\right)$$

where β_*j*–1_(Δ_*W*_) denotes the (*j* – 1)-st Betti number of the homology of Δ_*W*_. These expressions establish a direct connection between the topological invariants of the simplicial complex Δ and the algebraic invariants of its associated Stanley–Reisner ring.

#### *f*-Vectors and *h*-Vectors

Let Δ be a simplicial complex of dimension *d* – 1. The *f*-vector of Δ is defined as

11 $$\left(f_{0},f_{1},...,f_{d-1}\right)$$

where *f*_*i*_ denotes the number of *i*-dimensional faces of Δ. By convention, we set *f*_–1_ = 1 to account for the empty face.

The Hilbert series of the Stanley–Reisner ring *k*[Δ], also referred to as the Hilbert series of Δ, is given by

12 $$H_{\Delta }\left(s\right)=\sum_{d\geq 0}\dim_{k}\left(k{\left[\Delta \right]}_{d}\right)s^{d}$$

where *k*[Δ]_*d*_ denotes the degree-*d* component of the Z -graded ring *k*[Δ].

For a (*d* – 1)-dimensional simplicial complex Δ, it is a classical result that the Hilbert series can be expressed as a rational function of the form

13 $$H_{\Delta }\left(s\right)=\frac{h_{0}+h_{1}s+\cdot \cdot \cdot +h_{d}s^{d}}{{\left(1-s\right)}^{d}}$$

where (*h*_0_, *h*_1_, ···, *h*_*d*_) is the *h*-vector of Δ, or equivalently, of its Stanley–Reisner ring.

The *f*-vector and *h*-vector are related by the identity

14 $$\sum_{j=0}^{d}h_{j}s^{j}=\sum_{j=0}^{d}f_{j-1}{\left(1-s\right)}^{d-j}s^{j},\mathrm{with}\;f_{-1}=1$$

Equivalently, the entries of the *h*-vector can be expressed in terms of the *f*-vector by the relation

15 $$h_{j}=\sum_{i=0}^{j}{\left(-1\right)}^{j-i}\left(\begin{array}{l} d-i\\ j-i \end{array}\right)\;f_{i-1},j=0,1,...,d$$

and conversely, the *f*-vector can be recovered from the *h*-vector via

16 $$f_{j-1}=\sum_{i=0}^{j}\left(\begin{array}{l} d-i\\ j-i \end{array}\right)h_{i},j=0,1,...,d$$

#### Filtration and Persistent Stanley–Reisner Theory (PSRT)

A fundamental limitation of using a simplicial complex Δ to model data is that it typically captures topological or combinatorial information on a single scale, thereby omitting geometric details that may vary across scales. To address this limitation, one introduces a multiscale framework through the use of filtrations, leading to the theory of persistent homology, which identifies topological features that persist across a range of scales.

Persistent Stanley–Reisner theory follows a similar philosophy, extending combinatorial and algebraic invariants to a persistent setting. Let Δ be an abstract simplicial complex on a finite vertex set *V*. Given a monotone function g:Δ→R , that is,

τ⊆σ⇒g(τ)≤g(σ)

we define the induced filtration of Δ by

17 $$\mathrm{g̃}=\left(\Delta_{g}^{r}|r\in R\right)$$

where each subcomplex Δ_*g*_^*r*^ ⊆ Δ is defined as

$$\Delta_{g}^{r}≔\left\{\sigma \in \Delta |g\left(\sigma \right)\leq r\right\}$$

For notational simplicity, we may write Δ^*r*^ in place of Δ_*g*_^*r*^ when the context is clear.

Given a subset *W* ⊆ *V*, then if ${\left(\Delta^{r}\right)}_{r\in R}$ is a filtration of Δ, the induced filtration on the subcomplex Δ_*W*_ is given by

18 $$\Delta_{W}^{r}≔\Delta^{r}\cap \Delta_{W}\subseteq \Delta^{r\prime }\cap \Delta_{W}=\Delta_{W}^{r\prime }\;\mathrm{for}\;\mathrm{all}\;r\leq r^{\prime }$$

We define the persistent Stanley–Reisner graded Betti number β_*i*,*i* + *j*_^*r*,*r*′^(*k*[Δ]) as

19 $$\beta_{i,i+j}^{r,r\prime }\left(k\left[\Delta \right]\right)=\sum_{\begin{array}{l} W\subseteq V\\ \left|W\right|=i+j \end{array}}\dim_{k}\left(\iota_{j-1}^{r,r\prime }:{\mathrm{H̃}}_{j-1}\left(\Delta_{W}^{r};k\right)\to {\mathrm{H̃}}_{j-1}\left(\Delta_{W}^{r\prime };k\right)\right)$$

where ι_*j*–1_^*r*,*r*′^ is the homomorphism induced by the inclusion Δ_*W*_^*r*^ → Δ_*W*_^*r*′^ on the (*j* – 1)-st reduced homology, and β_*j*–1_(Δ_*W*_^*r*^) is the persistent Betti number of the inclusion from Δ_*W*_^*r*^ to Δ_*W*_^*r*′^. Summing over all relevant subsets, *W* yields a multiscale refinement of Hochster’s formula, capturing the persistent homological features of the complex across varying levels of filtration.

The persistent Stanley–Reisner graded Betti numbers β_*i*,*i*+*j*_^*r*,*r*′^(*k*[Δ]) generalize classical persistent Betti numbers; for example,

$$\beta_{i,\left|V\right|}^{r,r\prime }=\beta_{\left|V\right|-i-1}^{r,r\prime }$$

and further encode additional combinatorial information by tracking all monomial degrees in the resolution.

One may also extend combinatorial invariants such as *f*-vector and *h*-vector to persistent settings. The persistent *h*-vector is defined by

20 $$h_{m}^{r,r\prime }=\sum_{j=0}^{m}\left(\begin{array}{l} n-d+m-j-1\\ m-j \end{array}\right)\left(\sum_{i=0}^{j}{\left(-1\right)}^{i}\beta_{i,j}^{r,r\prime }\right)$$

and the corresponding persistent *f*-vector is given by

21 $$f_{m-1}^{r,r\prime }=\sum_{i=0}^{m}\left(\begin{array}{l} d-i\\ m-i \end{array}\right)h_{i}^{r,r\prime },m=0,1,...,d$$

In the case *r* = *r*′, the persistent *f*-vector coincides with the classical Stanley–Reisner *f*-vector; that is, *f* ^*r*,*r*′^ = *f*. When the underlying simplicial complex is large, the filtration often yields a substantial number of faces, leading to rapid growth in the components of the *f*-vector. To normalize this growth and facilitate meaningful comparisons across scales, we define the average rate of the *f*-vector by

$$\mathrm{f̅}\left(r\right)≔\frac{f^{r,r}}{r}$$

Finally, consider a filtration ${\left(\Delta^{r}\right)}_{r\in R}$ of the simplicial complex Δ. As *r* increases, the corresponding Stanley–Reisner ideals *I*(Δ^*r*^) evolve through the reverse inclusion

$$I\left(\Delta^{r\prime }\right)\subseteq I\left(\Delta^{r}\right)\;\mathrm{for}\;r\leq r^{\prime }$$

Let $P\left(\Delta^{r}\right)$ denote the set of facet prime monomial ideals (or facet ideals) of Δ^*r*^. For each *i* ≥ 0, define $P_{i}\left(\Delta^{r}\right)$ to be the subcollection of $P\left(\Delta^{r}\right)$ consisting of those associated with *i*-dimensional facets. Then, we have the disjoint union

$$P\left(\Delta^{r}\right)=\overset{\dim\left(\Delta^{r}\right)}{\underset{i=0}{\cup }}P_{i}\left(\Delta^{r}\right)$$

This decomposition motivates a persistent interpretation of the evolution of facet ideals over the filtration. In analogy to persistent homology, we refer to the facet ideals *P*_σ_ of *I*(Δ^*r*^) as the persistent facet ideals of Δ, representing combinatorial structures that persist across filtration levels.

We define the facet persistence Betti number β_*i*_^*r*,*r*′^ to be the number of the persistent facet ideals in $P_{i}\left(\Delta^{r}\right)$ that are persistent facet ideals in $P_{i}\left(\Delta^{r\prime }\right)$ . Explicitly,

$$\beta_{i}^{r,r\prime }=\left|P_{i}\left(\Delta^{r}\right)\cap P_{i}\left(\Delta^{r\prime }\right)\right|$$

Analogously to the case of persistent *f*-vectors, we define the average rate of the facet persistence Betti numbers by

$$\mathrm{\beta ̅}\left(r\right)≔\frac{\beta^{r,r}}{r}$$

### Vectorization of Persistent Commutative Algebra

#### Commutative Algebra on Bipartite Complexes

In (metallo)­protein–ligand binding interactions, bipartite complexes refer to molecular assemblies composed of two distinct, nonoverlapping (metallo)­protein and ligand components that interact to form a functional unit. In graph theory, a bipartite complex refers to a structure where vertices (or nodes) are divided into two distinct, disjointed sets, and edges only connect vertices from different sets. This bipartite structure is adopted in our commutative algebra analysis of (metallo)­protein–ligand binding data.

#### Element-Specific Commutative Algebra

There are various types of intramolecular and intermolecular interactions, including hydrogen bonding, electrostatic forces, and hydrophobic and hydrophilic interactions. To effectively characterize these critical interactions, element-specific modeling was developed in our earlier work,^37^ demonstrating notable effectiveness. In particular, molecular interactions enriched within various pairwise combinations of atom sets are captured by using persistent commutative algebra (PCA) modeling and subsequently represented through PCA-based vectorization.

**Table 3:** Details of the Data Sets Utilized for Benchmark Tests in This Study

data set	total	training set	test set
PDBbind-v2016 ^39^	4057	3767	290
metalloprotein–ligand^51^	2463	1845	618

For the PDBbind-v2016 data set, molecular interactions are characterized based on four commonly occurring atom types in proteins: carbon (C), nitrogen (N), oxygen (O), and sulfur (S), along with ten atom types in ligands, including carbon (C), nitrogen (N), oxygen (O), sulfur (S), phosphorus (P), fluorine (F), chlorine (Cl), bromine (Br), iodine­(I), and hydrogen (H). In the case of the metalloprotein–ligand data set, additional atomic interactions involving metal ions must be considered. Based on a statistical analysis of the metal ions frequently occurring in protein–ligand binding pockets, we include seven additional atom types: zinc (Zn), magnesium (Mg), manganese (Mn), calcium (Ca), sodium (Na), iron (Fe), and nickel (Ni) due to their prevalent presence. Table 4 gives the counts of some frequently occurring metal atoms in the training and test sets in the metalloprotein–ligand binding affinity data set. Cu atoms were excluded in our machine-learning model due to their infrequent occurrence in metalloprotein–ligand complexes.

**Table 4:** Counts of Some Frequently Occurring Metal Ions in the Training and Test Sets in the Metalloprotein-Ligand Binding Affinity Data Set

	elements							
data set	Zn	Mg	Ca	Mn	Ni	Fe	Na	Cu
training set	1389	614	516	319	63	60	48	8
test set	458	220	163	114	18	15	12	7

For protein–ligand complexes in the PDBbind-v2016 data set, it is sufficient to consider only protein–ligand interactions. However, for the metalloprotein–ligand binding affinity prediction, three types of interactions must be taken into account: protein–ligand interactions, metal ion–ligand interactions, and protein–metal ion interactions. Specifically, there are 4 × 10 = 40 atom pair combinations for protein–ligand interactions (P–L), 7 × 4 = 28 combinations for metal ion–protein interactions (M–P), and 7 × 10 = 70 combinations for metal ion–ligand interactions (M–L). Consequently, 40 types of atomic interactions are considered for protein–ligand complexes in the PDBbind-v2016 data set, while a total of 138 interaction types are incorporated for metalloprotein–ligand complexes.

For the 3D coordinate point cloud corresponding to each atom group combination, we utilize PCA to generate vectorized representations. The facet-persistent Betti numbers and their corresponding rates are employed to design a set of features for each atom group. A cutoff distance of 12 Å from the ligand is used to collect nearby protein atoms, while a cutoff distance of 15 Å from the ligand is applied to gather metal atoms. In the PCA-based featurization process, the filtration range is set from 1 Å to 12 Å or 15 Å for each case, with a step size of 0.5 Å for the filtration steps.

Rips complexes are constructed for each point cloud, while bipartite complexes are generated to capture the three types of molecular interactions. In our implementation, we focus exclusively on the facet Betti numbers and their corresponding rates for 0 and 1-simplices. The final molecular descriptor is obtained by concatenating the PCA-derived feature vectors from each point cloud. Figure 4 presents a schematic illustration of the general persistence Betti curves computed for a representative set of atoms.

#### Category-Specific Commutative Algebra

In addition to element-specific modeling, we also adopted a category-specific strategy to capture intrinsic molecular interactions characterized by amino acid types. Proteins are composed of 20 common amino acid residues, which can be classified into four major categories based on their side chain properties: hydrophobic (*H*), uncharged (*U*), negatively charged (*N*), and positively charged (*P*). We denote the atoms belonging to these categories as *A*_*H*_, *A*_*U*_, *A*_*N*_, and *A*_*P*_, respectively. Table 5 summarizes the four categories along with their corresponding amino acid types. Figure 3 shows the distribution of average atom numbers within different amino acid categories for the proteins in the PDBbind-v2016 and metalloprotein–ligand binding affinity data sets. Protein atoms within a 12 Å cutoff distance from the ligand were selected for atom count analysis.

**Table 5:** Four Categories of Amino Acid Residues in Proteins According to Their Side Chain Properties

hydrophobic (*H*)	uncharged (*U*)	negatively charged (*N*)	positively charged (*P*)
glycine (Gly)	serine (Ser)	aspartic (Asp)	lysine (Lys)
alanine (Ala)	threonine (Thr)	glutamic (Glu)	arginine (Arg)
valine (Val)	asparagine (Asn)		histidine (His)
leucine (Leu)	glutamine (Gln)		
isoleucine (Ile)	tyrosine (Tyr)		
methionine (Met)	cysteine (Cys)		
proline (Pro)			
phenylalanine(Phe)			
tryptophan (Trp)			

**Figure 3:**
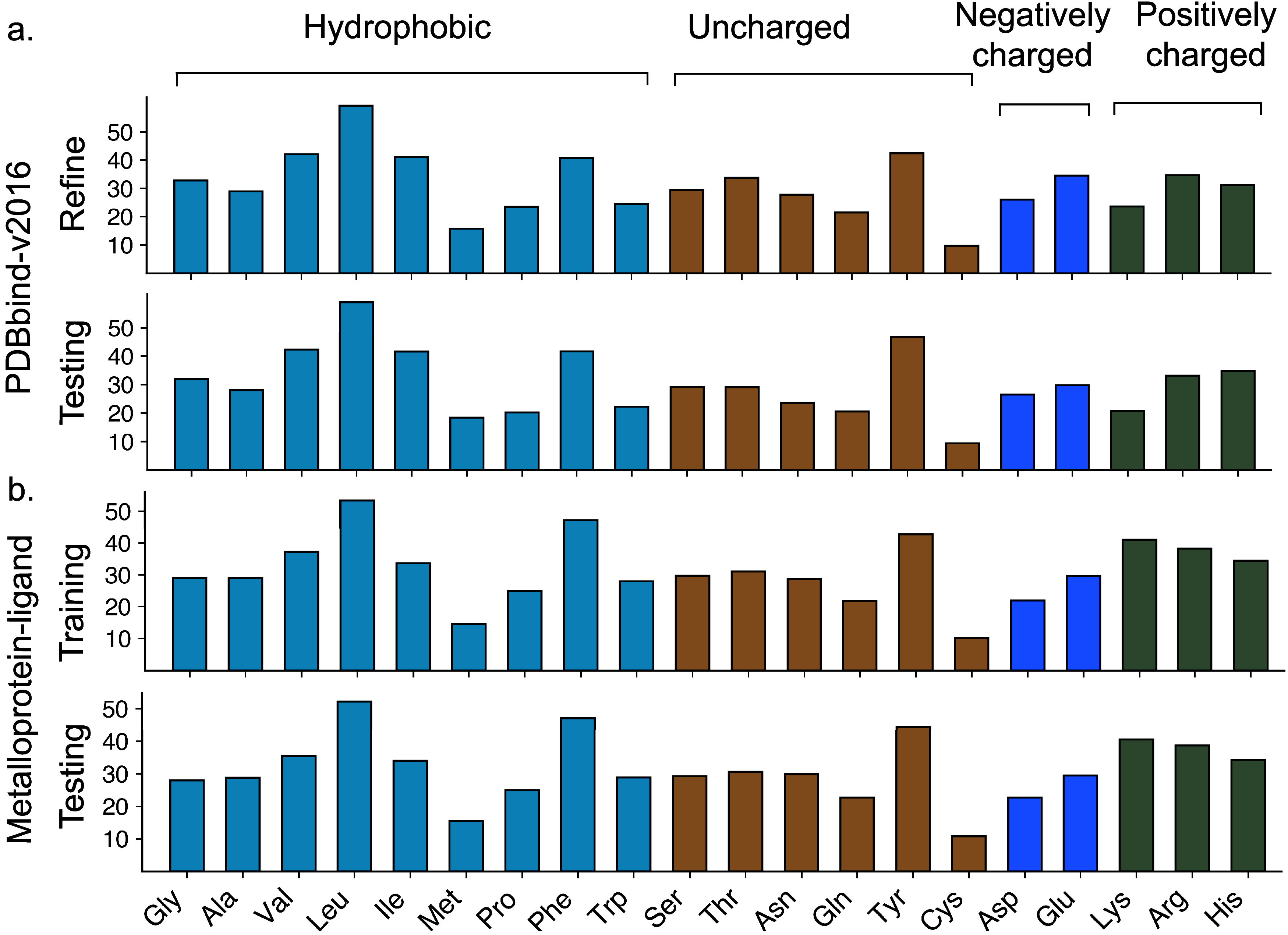
(a) Average atom counts for different amino acid types across those protein–ligand complexes in the refine and test sets of the PDBbind-v2016 data set. (b) Average atom counts for different amino acid types across those protein–ligand complexes in the training and testing sets of the metalloprotein–ligand binding affinity data sets. Protein atoms within a 12 Å cutoff distance from the ligand were selected for the atom count analysis.

As with the element-specific atom groupings used for ligands and metal ions, we consider 10 atom groups in ligands and 7 atom groups in metal ions based on their element types. Consequently, there are 40 atom group combinations in general protein–ligand complexes and 138 combinations in metalloprotein–ligand complexes. Similar PCA-based vectorizations are applied to these atom group combinations to generate molecular descriptors.

### Natural Language Processing (NLP) Molecular Descriptors

Natural language processing (NLP) has recently also become a popular machine-learning technique for molecular biosciences. We utilized NLP techniques to boost the performance of our PSRT-based machine-learning models for protein–ligand binding affinity predictions. Different from our PSRT theory, which analyzes molecular 3D structures, NLP extracts molecular physicochemical properties by analyzing molecular sequence patterns. For the protein–ligand complex, we have amino acid sequences for the protein and SMILES strings for the ligand. Some NLP-based molecular descriptors are designed using transformer techniques for both protein and ligand in works.^54,55^ By concatenating molecular descriptors from those pretrained deep learning models for protein sequence and ligand SMILES strings, we obtained a sequence representation for the protein–ligand complex.

#### Transformer-Based Protein Language Model

The ESM-2 transformer model, introduced by Rives et al.,^54^ has become one of the most widely adopted protein language models, with applications in protein engineering and drug discovery. This model was trained on a data set containing 250 million amino acid sequences and employs a deep learning architecture with 34 layers and 650 million parameters. In this work, we utilized the ESM-2 model to generate sequence embeddings for proteins. At each layer, a sequence of length *L* is encoded into a matrix of size 1280 × *L*, excluding the start and end tokens. We extracted the sequence representation from the final (34th) layer and computed the average along the sequence length axis, resulting in a 1280-dimensional feature vector.

#### Transformer-Based Small Molecular Language Model

A transformer-based deep learning framework was introduced to extract molecular representations,^55^ serving as a powerful tool for machine-learning applications involving small molecules.^56^ This model was trained on a collection of over 700 million SMILES strings obtained from databases such as ChEMBL, PubChem, and ZINC. Three pretrained variants were developed: model-C, model-CP, and model-CPZ. In the current study, we utilize the model-CPZ to generate molecular descriptors for ligands. For each ligand, the model produces a matrix of size 256 × 512, where 256 corresponds to the symbols representing the molecule and 512 is the dimension of the embedding vector for each symbol. The final molecular descriptors are obtained by the first vector among the 256 embedding vectors, resulting in a fixed-length feature vector.

### Machine Learning Modeling

We employ the Gradient Boosting Decision Tree (GBDT) algorithm to develop our machine-learning models using the Python scikit-learn package (v1.3.2) for implementation. GBDT is well-regarded for its robustness against overfitting, relative insensitivity to hyperparameter settings, and ease of implementation. The algorithm creates multiple weak learners or individual trees by bootstrapping training samples and integrates their outputs to make predictions. Although weak learners are prone to making poor predictions, the ensemble approach can reduce overall errors by combining the predictions of all of the weaker learners. We input resulting PSRT molecular descriptors and transformer-based molecular descriptors into the GBDT algorithm to build regression models, respectively. The GBDT hyperparameters used for modeling are listed in Table 6.

**Table 6:** Hyperparameters Used for Building Gradient Boosting Regression Models^a^

no. of estimators	max depth	min. sample split	learning rate
20,000/30,000	7	5	0.002
max features	subsample size	repetition	
square root	0.8	20 times	

a Tree numbers are set to be 20,000 and 30,000 respectively for PSRT and transformer-based molecular descriptor modeling.

### Evaluation Metrics

To quantitatively evaluate the performance of our binding affinity prediction models, we employ the Pearson correlation coefficient (PCC), defined as

22 $$\mathrm{PCC}\left(x,y\right)=\frac{\sum_{m=1}^{M}\left(y_{m}^{e}-{\mathrm{y̅}}^{e}\right)\left(y_{m}^{p}-{\mathrm{y̅}}^{p}\right)}{\sqrt{\sum_{m=1}^{M}{\left(y_{m}^{e}-{\mathrm{y̅}}^{e}\right)}^{2}\sum_{m=1}^{M}{\left(y_{m}^{p}-{\mathrm{y̅}}^{p}\right)}^{2}}}$$

where *y*_*m*_^*e*^ and *y*_*m*_^*p*^ denote the experimental and predicted binding affinity values for the *m*th sample, respectively, and *y̅* ^*e*^ and *y̅* ^*p*^ are their corresponding mean values.

We also report the root mean squared error (RMSE), which is computed as

$$\mathrm{RMSE}=\sqrt{\frac{1}{M}\sum_{m=1}^{M}{\left(y_{m}^{e}-y_{m}^{p}\right)}^{2}}$$

where *y*_*m*_^*e*^ and *y*_*m*_^*p*^ represent the experimental and predicted binding affinity values for the *m*th sample, respectively.

We employ the above two metrics to assess the performance of our machine-learning models on both data sets. The original labels for these data sets are given as p*K*_d_ values, which can be converted to binding free energies (in kcal/mol) by multiplying by a constant factor of 1.3633. Our models achieve low RMSE values across both data sets. For the PDBbind-v2016 data set, we convert the labels to binding energies and use them for RMSE comparisons with previously published models.

## Model Interpretability

Our persistent Stanley–Reisner approach offers excellent interpretability for modeling molecular structures, motivating us to apply it in the design of molecular descriptors. As an illustrative example, we consider the vectorization of compound C_60_, as shown in Figure 4. The molecule C_60_ consists of 60 carbon atoms arranged in a truncated icosahedron—a polyhedral structure composed of 12 pentagonal and 20 hexagonal faces—resembling the shape of a soccer ball. In this configuration, each pentagon is surrounded by six hexagons. There are two types of bonds in the molecule. Single bonds, with a bond length of 1.453 Å, are located between pentagons and hexagons, totaling 60 in number. Double bonds with a bond length of 1.367 Å are found between pairs of hexagons. Since each hexagon is adjacent to three hexagons and three pentagons, there are 20 × 3/2 = 30 double bonds in total. Each atom in C_60_ forms two single bonds and one double bond, connecting it to three neighboring atoms.

**Figure 4:**
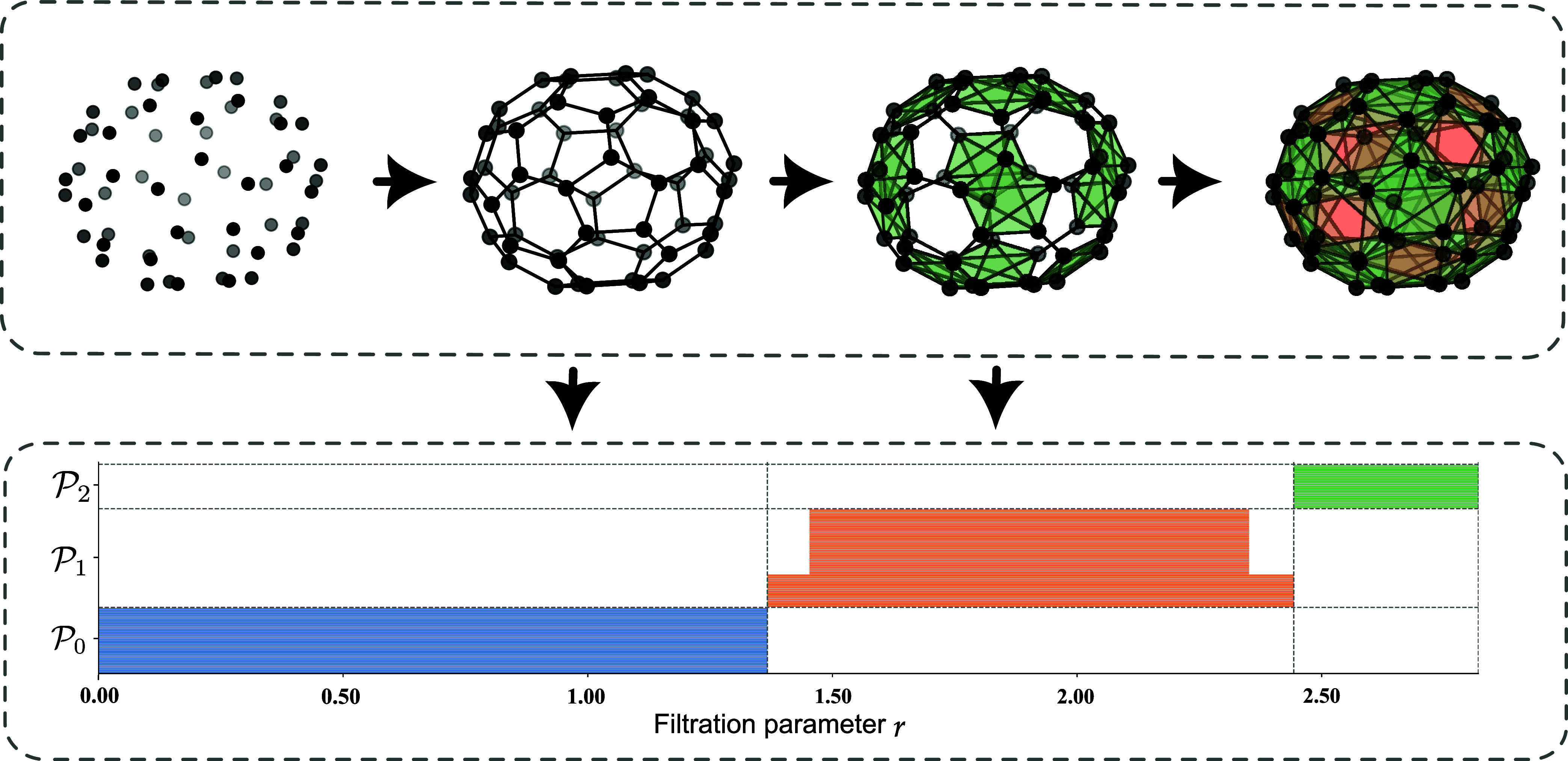
Illustration of the filtration process in the persistent commutative algebra. Given a point cloud input (a C_60_ molecule), a corresponding simplicial complex with associated filtration is constructed (upper panel). Facet persistence barcodes are then computed across various dimensions (lower panel).

The figure displays the Vietoris–Rips filtration of the molecule along with the corresponding barcodes associated with its persistent facet ideals. A notable geometric feature is captured within the interval [1.3, 1.5]. There are 60 persistent ideal bars in the $P_{0}$ panel corresponding to the 60 atoms. These bars vanish simultaneously around filtration parameter *r* = 1.367 Å because each vertice (atom) merges into an edge (double bond). In the $P_{1}$ panel, 30 bar (edges) show up at *r* = 1.367 Å, corresponding to double bonds. These bars merge into triangles, which are formed due to new edges connecting a vertex *v*_*i*_ to *v*_*i*+2_ under a cyclic ordering of the hexagonal vertices. These new edges have length of 2.443 Å, which corresponds to the larger ending values of these orange bars. Such persistent facet ideals explains the existence of these 30 edges. A set of 60 pars appears at filtration *r* = 1.453 Å corresponding to these single bonds. These edges persist until new edges are created to connect vertex *v*_*i*_ to *v*_*i*+2_ under a cyclic ordering of the pentagonal vertices, where new edges have a length of 2.352 Å. This accounts for the observed variation in the birth times of persistent facet ideals corresponding to these edges, as shown in the $P_{1}$ panel. Finally, $P_{2}$ shows the birth and death of triangles in the system. Therefore, facet persistence barcodes reveal the geometric connectivity of molecular structures and are interpretable, making commutative algebra a rational approach for machine learning.

In addition, the element- and category-specific approaches used in this work capture key intramolecular and intermolecular interactions that play crucial roles in determining the chemophysical properties of biomolecules. These essential interactions include hydrogen bonding, hydrophobic and hydrophilic interactions, van der Waals forces, electrostatic forces, and others. For example, C–C specific atoms primarily contribute to hydrophobic interactions, while N–N and N–O specific atoms are associated with hydrogen bonding, and O–C interactions often reflect electrostatic effects. In particular, these interactions exhibit different distance-dependent strengths. To effectively capture these critical interactions, we integrate persistent Stanley–Reisner complexes with various atom groupings. Altogether, this analysis offers a highly interpretable insight into the molecular structure, demonstrating the suitability of persistent facet ideals for molecular description and characterization and improving the performance of machine-learning models.

Persistent homology^35^ and persistent Laplacian theory^25^ have become popular and competitive approaches in machine learning modeling. While our persistent commutative algebra shares similarities with persistent homology in terms of a simplicial complex, it captures data patterns from a distinct perspective. Persistent homology focuses on the persistence of topological invariants (such as points, loops, and cavities), providing global structural information across the filtration process. In contrast, our persistent commutative algebra reveals more intrinsic local properties of simplices of various dimensions (including points, edges, and triangles) throughout the filtration. Compared to persistent Laplacian theory,^25,57^ our method also demonstrates greater computational feasibility. Persistent Laplacian theory extracts data patterns based on the eigenvalues of Laplacian matrices, a process that is computationally demanding. Ultimately, each approach offers unique strengths and may be better suited to different types of data and tasks.

## Conclusions

As a foundational part of algebraic geometry and algebraic number theory, commutative algebra studies polynomial rings, their ideals, and the modules over such rings. However, commutative algebra has rarely been applied to data science and machine learning. The persistent Stanley–Reisner theory (PSRT),^31^ introduced by Suwayyid and Wei, offers a new opportunity to develop commutative algebra machine learning (CAML) and commutative algebra deep learning (CADL) for data. Stanley–Reisner theory, also known as face ring theory, creates a profound connection between combinatorics and commutative algebra. PSRT integrates tools from algebra, combinatorics, and multiscale analysis (i.e., filtration) to study simplicial complexes via Stanley–Reisner rings.

This work proposes CAML for data analysis. We pair PSRT with a robust machine-learning method, gradient-boosted decision trees (GBDT), which utilize an ensemble of decision trees to make predictions. GBDT is known for its high accuracy and efficiency, particularly for relatively small data sets that are not suitable for deep learning algorithms. We consider two biomolecular data sets, i.e., a protein–ligand binding data set (PDBbind-v2016) and a metalloprotein–ligand binding data set, to validate the proposed CAML model. Due to the intricate interactions in (metallo)­protein–ligand complexes, we propose new algorithms, such as commutative algebra on a bipartite complex, element-specific commutative algebra, and category-specific commutative algebra, to capture the physics and chemistry underlying the interactions. The performance of the proposed CAML model is compared to other state-of-the-art methods in the literature. We demonstrate that CAML is an extremely promising new method for protein–ligand binding predictions.

Protein–ligand binding prediction serves as a case study of the proposed CAML model. CAML can easily be applied to other biomolecular data predictions and general problems in science and engineering. We believe that proposed commutative algebra learning (CAL) represents an emerging direction in machine learning and data science.

## Data Availability

All data and the code needed to reproduce this paper’s result can be found at https://github.com/WeilabMSU/CAML. For detailed information on the metalloprotein–ligand complex data set, please refer to the reference.^40^ The PDBbind-v2020 data set is available at http://pdbbind.org.cn/.

## °

School of Mathematics, Georgia Institute of Technology, Atlanta, Georgia 30332, United States
